# Status and Associated Factors of Breakfast Consumption Among Chinese Residents: A National Cross‐Sectional Study

**DOI:** 10.1002/fsn3.70136

**Published:** 2025-05-26

**Authors:** Ming Liu, Shujie Dong, Yifan Li, Shaolin Liang, Chun Kai Leung, Casper J. P. Zhang, Sicun Li, Yibo Wu, Wai‐kit Ming

**Affiliations:** ^1^ Department of Infectious Diseases and Public Health, Jockey Club College of Veterinary Medicine and Life Sciences City University of Hong Kong Hong Kong China; ^2^ Institute for Six‐Sector Economy Fudan University Shanghai China; ^3^ Global Society and Sustainability Lab, Faculty of Social Sciences The University of Hong Kong Hong Kong China; ^4^ Institute for the Humanities and Social Sciences The University of Hong Kong Hong Kong China; ^5^ School of Public Health The University of Hong Kong Hong Kong China; ^6^ Department of Social Medicine and Health Education, School of Public Health Peking University Beijing China; ^7^ Institute of Global Governance and Innovation for a Shared Future, City University of Hong Kong, Hong Kong, China

**Keywords:** breakfast, Chinese, cross‐sectional study, factor, logistic regression

## Abstract

While the importance of breakfast for human health is widely acknowledged, there is limited understanding of the factors influencing breakfast habits among Chinese residents. We conducted a nationwide cross‐sectional survey between June 20 and August 31, 2022, analyzing weekly breakfast frequency, food categories, and associated factors to daily breakfast consumption using multivariable logistic regression, with subgroup analyses by gender and residence (urban or rural). Among 21,875 participants, 41.0% reported non‐daily breakfast consumption. Common breakfast items included staples like rice, wheat, and corn (70.5%), eggs (56.2%), dairy products (42.0%), and soy drinks (36.8%), while less frequently consumed items included meat products (26.3%), potatoes (23.4%), fresh vegetables and fruits (20.2%), and pickled vegetables (18.2%). Behavioral factors such as sleeping 6–7 h and abstaining from smoking and sugar‐sweetened beverages, along with health factors like better quality of life, family health, and higher self‐efficacy, were positively associated with daily breakfast consumption. Depression was negatively associated. Sociodemographic factors including female gender, living in southern China, and having children were positively associated, whereas rural residency, higher education levels, being a student or unemployed, and living alone were negatively associated. Subgroup analyses revealed pronounced variations in breakfast habits by residence (urban vs. rural) but fewer differences by gender. Non‐daily breakfast consumption is prevalent among Chinese residents, with multiple sociodemographic, behavioral, and health factors influencing this behavior. Region‐ and gender‐specific strategies are essential to promote healthy breakfast habits, address disparities, and encourage healthier breakfast practices across diverse subgroups.

## Graphical abstract

This nationwide cross‐sectional study analyzed breakfast consumption patterns among 21,875 Chinese residents. Findings revealed that 41.0% were non‐daily breakfast consumers, with common breakfast items including rice, wheat, and corn. Behavioral and health factors, such as adequate sleep and better family health, were positively associated with daily breakfast consumption, while sociodemographic disparities were evident between rural and urban residents. Tailored region‐ and gender‐specific interventions are necessary to promote healthier breakfast habits and address disparities across subgroups.

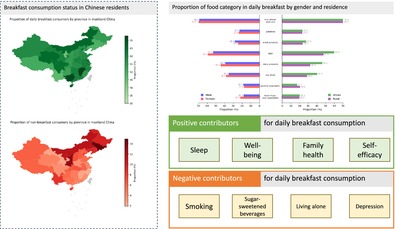

## 1 Introduction

Breakfast, commonly considered one of the most important meals of the day, has been shown to play a significant role in controlling weight, reducing cardio‐metabolic risks, and improving cognitive performance in recent years (Gibney et al. 2018; Takagi et al. 2019; Kawabata et al. 2021). Regular breakfast consumption may also help promote overall healthy eating habits (St‐Onge et al. 2017). Eating breakfast regularly is recommended as an integral component of a nutritionally optimal diet by many professional societies and associations (British Dietetic Association n.d.; Dietitians Association of Australia n.d.; American Academy of Nutrition and Dietetics n.d.; Society CN 2022). Previous national dietary surveys explored patterns of breakfast consumption in European countries, the United States, and Canada and revealed that skipping breakfast is a common phenomenon across different countries (Gibney et al. 2018). For instance, a survey conducted in 39 European states showed that about one third to half of children did not have breakfast every day (Currie et al. 2012a, 2012b). A survey in Canada showed that up to 10% of children and 11% of adults were breakfast skippers (Barr et al. 2013). Although dietary advice advocating regular breakfast consumption has been issued by the Chinese government and strongly recommended in the Chinese Dietary Guidelines, it is estimated in a provincial survey that approximately 45% of adolescents did not consume breakfast daily (Wang et al. 2016). Besides, for consumption patterns, there have been significant changes in Chinese adults, characterized as a shift from a high‐carbohydrate diet to a high‐fat diet (Huang et al. 2021). Currently, data regarding breakfast frequency and eating patterns for both adolescents and adults are still lacking at the national level in China.

Eating breakfast is a complex issue because eating behavior is multifaceted and affected by many factors to varying degrees. Previous studies have analyzed factors influencing healthy eating behaviors (Sogari et al. 2018; Lai et al. 2021), including behavioral factors (e.g., lack of knowledge and cooking skills, lack of time, community programs, family support, communication with health professionals, low accessibility to healthy foods, taste preferences, physical limitations, product prices (Lai et al. 2021; Ryan et al. 2022; Aboueid et al. 2019; Camp‐Spivey et al. 2022)), sociodemographic (e.g., age, marital status, education, employment (Lai et al. 2021; Ryan et al. 2022; Aboueid et al. 2019; Camp‐Spivey et al. 2022), financial constraints (Hovdenak et al. 2024)), health factors (e.g., family health, anxiety, depression (Ishikawa et al. 2018; Aucoin et al. 2021; Zahedi et al. 2022), waist circumference (Jigeer et al. 2024)), and even pandemics (Kadir et al. 2024). However, these studies mainly focus on overall daily food intake rather than a specific meal, breakfast, and the findings cannot be simply extrapolated to breakfast. Moreover, factors facilitating breakfast eating behaviors remain unclear, and targeted interventions are still lacking in China. To provide a comprehensive explanation framework, various behavioral, sociodemographic, and health‐related features should be taken into account when examining factors related to health behaviors. Furthermore, gender‐based differences in dietary habits, preferences, and nutritional strategies are well‐documented considerations (Turrell 1997; Grzymisławska et al. 2020). Similarly, regional variations in China, such as differences in income levels, infrastructure, and available food, may contribute to diverse breakfast consumption patterns (Tian 2019; Ren et al. 2021).

Till now, no previous studies have revealed the overall status and associated factors of breakfast consumption patterns at the national level in China. To address these issues, this study aims to conduct a nationwide quantitative survey to evaluate the current status of breakfast consumption among Chinese residents and investigate the associated factors, with a specific focus on how these factors vary across gender and region. This approach will provide a comprehensive overview of breakfast consumption patterns in China and identify key areas for intervention to promote healthier eating habits.

## 2 Materials and Methods

### 2.1 Study Design and Participants

The cross‐sectional survey was conducted from June 20 to August 31, 2022, across 148 cities in mainland China, covering 23 provinces, five autonomous regions, and four municipalities. A multistage stratified sampling strategy was implemented through five administrative tiers: provincial → municipal → district/country→ township/town/sub‐district→ community/village. The minimum sample requirement per province‐level division was determined according to population proportions derived from the Seventh National Census data, with stratified thresholds of 500, 1000, 1500, 2000, and 2500 participants assigned to different population strata. Non‐proportional quota sampling was adopted at the individual level to ensure a robust representation of demographic subgroups based on gender and age attributes. The sampling method can be seen in Figure S1. The research protocol was proved by the Ethics Committee of the Health Culture Research Centre of Shaanxi, China, and has been officially registered in the China Clinical Trial Registry (Registration No: ChiCTR2200061046) (“Psychology and Behaviour Investigation of Chinese Residents (PBICR) in 2022”, 2022). The comprehensive overview of PBICR is provided in previous studies (Yang et al. 2024; Wu et al. 2024).

Provincial responsible persons of investigators were recruited through the online release of the notice, and respondents were recruited through posters, paper, or electronic recruitment notices by investigators. Data collection involved face‐to‐face interviews whenever possible. In communities where investigators could conduct on‐site investigations, electronic questionnaires were distributed one‐on‐one and administered on the spot. However, due to the COVID‐19 epidemic, alternative methods were employed when face‐to‐face investigations were unavailable. In such cases, investigators utilized instant communication tools like WeChat to distribute electronic questionnaires and conducted online video investigations through Tencent Meeting and WeChat videos. This approach allowed participants to interact with investigators in real‐time, ensuring that the communication and data collection processes were consistent across both methods, thereby minimizing potential differences. Before data collection, informed consent was obtained from all respondents. The investigators also determined that the respondent meets the inclusion criteria and does not meet the exclusion criteria for respondents. Data were collected using the ‘www.wjx.cn’ platform, with strict measures in place to ensure the anonymity and confidentiality of personal information (Wang et al. 2022a).

Inclusion criteria for participants in this study were as follows: (a) age of 12 years or older; (b) nationality of the People's Republic of China; (c) permanent resident status in China with an annual departure time of no more than 1 month; (d) voluntary participation in the study and completion of the informed consent form; (e) ability to independently or with the help of investigators complete the questionnaire survey; (f) sufficient comprehension of each item in the questionnaire.

Exclusion criteria for participants were as follows: (a) questionnaires completed in less than 240 s; (b) questionnaires with inconsistent logical responses; (c) questionnaires with missing information; (d) repeatedly filled questionnaires; (e) questionnaires containing identical or repetitive responses; (f) regular questionnaires with predefined or uniform responses.

### 2.2 Questionnaire

The questionnaire inquired about breakfast consumption patterns, including the weekly breakfast eating frequency (never; 1–2 times;3–4 times; 5–6 times; daily) and categories of foods consumed in daily breakfast (rice, wheat, and corn; potatoes; meat products; eggs; dairy products (e.g., milk, milk powder, milk beverages); soy drink; pickled vegetables; fresh fruits and vegetables) in the past year. The choice of these breakfast categories was based on The Chinese Dietary Guidelines (2022), (Society CN 2022). Respondents were categorized into two groups based on their reported weekly breakfast eating frequency in the past year: daily and non‐daily breakfast consumers. Daily breakfast consumption has been widely recommended as a healthy dietary habit and public health suggestion (Papoutsou et al. 2014; Odegaard et al. 2013).

Daily breakfast consumers refer to individuals who reported consuming breakfast every day. Non‐daily breakfast consumers are those who indicated eating breakfast occasionally or reported never eating breakfast, i.e., non‐breakfast consumers.

The survey collected information, including (a) sociodemographic characteristics: age, height, weight, gender, residence, region, occupation status, income, employment status, marital status, and highest education level, (b) residential status (whether the participants were living alone, and whether they had children), (c) personal behavior: sleep duration, consumption of sugar‐sweetened beverages, smoking habits, drinking status, and dietary supplement use, and (d) health status: assessment of depression, quality of life, perceived social support, self‐efficacy, and family health using standardized scales.

The Patient Health Questionnaire‐9 (PHQ‐9) was employed for depression assessment, utilizing a scoring system ranging from 0 to 27. Higher scores on the PHQ‐9 indicated more severe depression, with score categories representing minimal, mild, moderate, moderately severe, and severe depression, ranging from 0–4, 5–9, 10–14, 15–19, to 20–27, respectively PHQ‐9 is a reliable and valid measure of depression severity and valid and efficient tool for screening depression in general Chinese population (Kroenke et al. 2001; Wang et al. 2014).

The 5‐item World Health Organization Well‐Being Index (WHO‐5) was utilized to measure the quality of life, using a scoring system that ranged from 0 to 25. Higher scores on the WHO‐5 indicated a better quality of life. It was recommended to administer the Major Depression (defined by International Classification of Diseases‐10 codes) Inventory where the raw score is below 13 or if the patient response is 0 to 1 on any of the five items. In accordance with this guideline, a score below 13 or any 0 to 1 response on the items is indicative of poor well‐being, while any other score is regarded as reflecting good well‐being (Psychiatric Research Unit n.d.). WHO‐5 has good internal consistency, validity, and proven effectiveness in both epistemological studies and clinical research among Chinese populations (Fung et al. 2022).

The 3‐item short form of the Perceived Social Support Scale (PSSS‐SF3) was utilized to measure perceived social support, with scores ranging from 3 to 21. The scores fell into three categories: 3 to 9 for low support, 10 to 15 for moderate support, and 16 to 21 for high support (Li et al. 2020). PSSS‐SF3 is suitable for large‐scale cross‐sectional studies in Chinese populations (Sun et al. 2024).

In addition, the 3‐item short form of the New General Self‐efficacy scale (NGSES‐SF3) was used to assess self‐efficacy, with scores ranging from 3 to 15, where higher scores indicated greater self‐efficacy (Chen et al. 2001). NGSES‐SF3 has demonstrated great reliability and validity among mainland Chinese residents (Wang et al. 2022b).

Finally, the Family Health Scale‐Short Form (FHS‐SF) was utilized to assess family health. Family health can be conceptualized as the comprehensive well‐being and functioning of a family unit, encompassing a range of interconnected factors that impact the overall health and quality of life of its members. This concept transcends individual health and extends to the overall health of the family as a cohesive unit. The total scores on this scale ranged from 0 to 10, with higher scores indicating better family health (Crandall et al. 2020). FHS‐SF has good reliability and validity, which are reliable and effective tools for assessing the health literacy of the Chinese population (Sun et al. 2024; Table S1).

### 2.3 Statistical Methods

We initially performed descriptive statistics on participant characteristics and compared health status indicators between daily and non‐daily breakfast consumers. Subsequently, we assessed the distribution of breakfast eating frequency across the entire population and stratified by gender, residence (urban or rural), and province. To further explore the breakfast consumption pattern, we conducted our initial statistical analysis by stratifying different impact factors, and we found a large difference in the daily breakfast consumption pattern stratified by gender and residence. To explore this difference in depth, we summarized the proportion of different food categories in daily breakfast consumption stratified by gender and residence.

We utilized binary logistic regression to identify factors associated with daily breakfast consumption. This analysis was conducted for the overall population and also when stratified by gender and residence. Categorical attribute data were transformed into dummy codes for use in the logistic regression analyses. During the logistic regression process, we first conducted exploratory analyses on each variable to handle outliers, missing values, or categorical variables with low frequencies. We then performed univariable analyses to explore the unadjusted associations between variables and daily breakfast consumption. Variables meeting a retention threshold (*p* < 0.25) were included in a base multivariable model alongside potential confounding factors considered, including gender, age, region, residence, income, BMI, marriage status, employment status, education, depression, and perceived social support. Collinearity was evaluated using variance inflation factors (VIF < 10 for all variables), followed by interaction terms assessment between gender, residence, and behavior factors, retaining those significant at *p* < 0.01. Then, we employed backward elimination to remove nonsignificant predictors (*p* > 0.05 exclusion criterion), except for variables classified as confounders. Confounders were identified if removing a variable altered the odds ratios (OR) of personal behaviors and health factors by > 10%; it was retained regardless of statistical significance. The Hosmer‐Lemeshow test was used to check the fitness of the model. Results were considered statistically significant at *p* < 0.05.

The study adheres to the STROBE‐Nut reporting guidelines (Table S3). All statistical analyses and data visualizations were conducted using STATA (version 17.0) and Python (version 3.9.13).

## 3 Results

**FIGURE 1:**
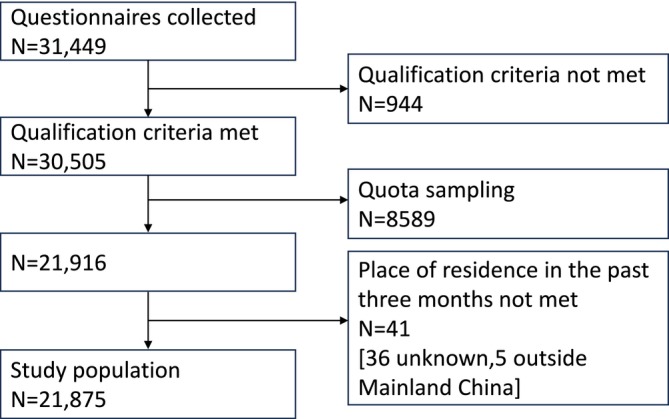
Flowchart of participant enrollment.

Of 31,449 questionnaires collected, 30,505 met the qualification criteria after logical checks (Appendix S1), and 21,916 remained after quota sampling based on population proportions from the seventh national census data (China NBoSo n.d.). 41 questionnaires were discarded because the place of residence in the past 3 months could not be obtained. Finally, 21,875 questionnaires were included in our final statistical analysis (Figure 1).

### 3.1 Breakfast Eating Behavior and Food Categories in Daily Breakfast

21,875 individuals were included in our analysis, of whom 12,913 (59.0%) were daily breakfast consumers, while 8962 (41.0%) were categorized as non‐daily breakfast consumers. Demographic characteristics are shown in Table 1. Compared to individuals who do not consume breakfast daily, those who have breakfast every day showed a significantly lower occurrence of moderate to severe depression, as well as poor well‐being and poor family health (Figure S2).

**TABLE 1:** Descriptive statistics on breakfast eating behavior among study participants.

Variables	*N* (%)
Total	21,875 (100.0)
Gender	
Male	10,933 (50.0)
Female	10,942 (50.0)
Per capita monthly income of households, yuan	
≤ 3000	7216 (33.0)
3001–6000	9005 (41.2)
> 6000	5654 (25.8)
BMI category, kg/m^2^,^a^	
Underweight (< 18.5)	3721 (17.0)
Normal (18.5 ≤ BMI < 24.0)	12,829 (58.6)
Overweight (24.0 ≤ BMI < 28)	4283 (19.6)
Obesity (≥ 28)	1042 (4.8)
Region	
North China	12,562 (57.4)
South China	9313 (42.6)
Place of residence in the past 3 months	
Urban	15,155 (69.3)
Rural	6720 (30.7)
Marital status	
Unmarried	8479 (38.8)
Married	12,417 (56.8)
Widowed or divorced	979 (2.6)
Highest education level	
Junior high or below	6960 (31.8)
Senior high school	5177 (23.7)
Undergraduate or above	9738 (44.5)
Employment status	
Employed	7583 (34.7)
Student	6566 (30.0)
Retired	2749 (12.6)
No fixed occupation	2608 (11.9)
Unemployed	2369 (10.8)
Whether having children	
No	9450 (43.2)
Yes	12,425 (56.8)
Whether living alone	
No	18,760 (85.8)
Yes	3115 (14.2)
Actual sleep time per night usually, hours	
< 5	1220 (5.5)
5–6	4488 (20.5)
6–7	8442 (38.6)
> 7	7745 (35.4)
Current or past smoking habit^b^	
Never smoked	17,885 (81.8)
Previously smoked but do not currently smoke	752 (3.4)
Currently smoking	3238 (14.8)
Current or past drinking status	
Never consumed alcohol	15,189 (69.4)
Previously consumed alcohol but do not currently consume	2144 (9.8)
Currently consume alcohol but did not consume in the past	1286 (5.9)
Consistently consume alcohol	3256 (14.9)
Weekly consumption of sugar‐sweetened beverages in the past year, bottles	
0	8397 (38.4)
≤ 3	9581 (43.8)
4–6	2794 (12.6)
≥ 7	1143 (5.2)
Dietary supplement behavior in the past year^c^	
No	12,699 (58.1)
Yes	9176 (41.9)
Perceived social support	
Low support	1689 (7.7)
Moderate or high support	20,186 (92.3)
Depression	
Minimal to mild depression	16,916 (77.3)
Moderate to severe depression	4959 (22.7)
Quality of life	
Poor well‐being	8684 (39.7)
Good well‐being	13,191 (60.3)
Family health	
Poor family health	5744 (26.2)
Moderate or excellent family health	16,131 (73.8)
Breakfast eating behavior	
Non‐daily breakfast consumer	8962 (41.0)
Daily breakfast consumer	12,913 (59.0)
Weekly breakfast eating frequency in the past year	
Never	1426 (6.5)
1–2 times per week	2052 (9.4)
3–4 times per week	2834 (13.0)
5–6 times per week	2650 (12.1)
Daily	12,913 (59.0)

^a^ BMI categories were defined according to Chinese classifications recommended by the health industry standards of the People's Republic of China: BMI ≥ 24 for overweight and BMI ≥ 28 for obesity (National Health Commission of the People's Republic of China n.d.).

^b^ Smoking includes regular cigarettes and electronic cigarettes.

^c^ Dietary supplements include protein, calcium, iron, zinc, multivitamins, vitamins A or D, DHA, and others (with an option to specify any additional supplements).

**FIGURE 2:**
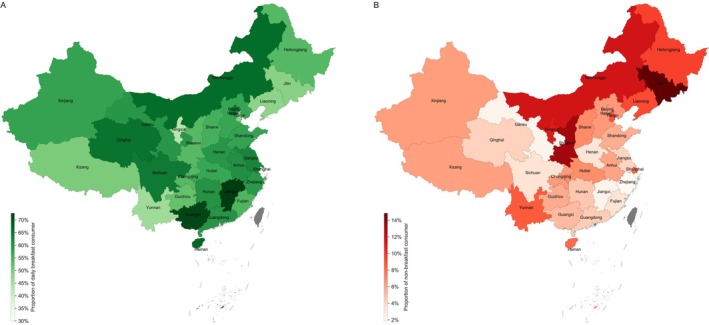
Proportion of (A) daily breakfast and (B) non‐breakfast consumers by province in mainland China.

Among the participants, 59.0% were identified as daily breakfast consumers, and 41.0% were identified as non‐daily breakfast consumers. A notable gender difference was observed, with a higher proportion of females (61.1%) being daily consumers compared to males (57.0%) (chi‐squared test, *p* = 0.0016 < 0.01). In terms of residence, urban residents exhibited a lower prevalence of non‐breakfast consumption (5.9%) compared to rural residents (7.9%) (chi‐squared test, *p* < 0.001) (Figure S3). Examining regional variations, the proportions of daily breakfast consumers were higher in Jiangxi (72.8%) and Guangxi (71.3%), while they were lower in Shanghai (30.0%) and Ningxia (42.8%). The prevalence of non‐breakfast consumption was notably higher in specific northern provinces, including Jilin (14.9%), Shaanxi (13.2%), and Ningxia (11.0%) (Figure 2).

Rice, wheat, and corn (70.5%), eggs (56.2%), dairy products (42.0%), and soy drink (36.8%) were common food categories in breakfast. Meat products (26.3%), potatoes (23.4%), fresh vegetables and fruits (20.2%) and pickled vegetables (18.2%) were less frequently consumed in daily breakfast. A higher proportion of males than females chose meat products at breakfast and on the other hand, a higher proportion of females ate eggs, dairy products, soy drink, and fresh vegetables at breakfast. The proportion of eggs, dairy products, and soy drink consumption in urban residents was much higher than that in rural residents (Figure S4).

**FIGURE 3:**
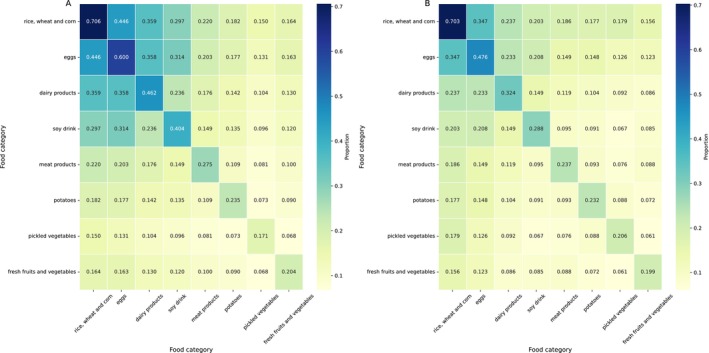
Combinations of Food Categories in Daily Breakfast Consumption among (A) Urban and (B) Rural Residents. The values displayed within each cell represent the relative proportion of individuals who selected the corresponding food combinations as part of their daily breakfast.

Chinese urban residents have a richer choice of breakfast categories than rural residents, with more combinations of food categories in their breakfast consumption. Notably, eggs (60.0%), dairy products (46.2%), and soy drink (40.4%) are frequently consumed by urban residents. Moreover, the combinations of these three food categories with other food categories were higher among urban residents compared with rural residents. In contrast, pickled vegetables, the least consumed category in urban areas, were consumed more frequently by rural residents during their daily breakfast (Figure 3).

### 3.2 Factors Associated With Daily Breakfast Consumption

The factors associated with daily breakfast consumption in the study are summarized in Table S2. Regarding behavioral factors, a U‐shaped relationship was observed between sleep duration and daily breakfast consumption, with both shorter (< 6 h) and longer (> 7 h) sleep durations associated with a reduced likelihood of daily breakfast consumption, especially among individuals with less than 5 h of sleep (OR: 0.36 [95% CI: 0.31–0.42]). Compared to individuals who never smoked, current smokers were less likely to consume breakfast daily (0.82 [0.72–0.93]), while individuals who had quit smoking were more likely to do so (1.49 [1.13–1.99]). A similar trend was observed with alcohol consumption: compared to those who never consumed alcohol, former drinkers were more likely to eat breakfast daily (1.32 [1.13 to 1.52]). Consumption of sugar‐sweetened beverages was inversely associated with daily breakfast consumption, irrespective of the quantity consumed. In contrast, dietary supplement use was positively associated with daily breakfast consumption (1.30 [1.20–1.40]).

As for health factors, obesity was positively associated with daily breakfast consumption (1.22 [1.00–1.48]), while depression showed an inverse relationship (0.69 [0.63–0.75]). Better quality of life (1.22 [1.12–1.34]), family health (1.82 [1.66–2.00]), and higher self‐efficacy (1.07 [1.05–1.09]) were all positively associated with daily breakfast consumption.

For sociodemographic factors, older age (1.01 [1.00–1.01]), female gender (1.21 [1.11–1.33]), and residents in south China (1.32 [1.22–1.43]) were associated with daily breakfast consumption. Having children (1.75 [1.50–2.05]) was also positively linked to daily breakfast consumption. Conversely, rural residents (0.70 [0.61–0.82]), higher education levels (undergraduate or above (0.76 [0.68–0.85])), being a student or without stable employment, and living alone (0.84 [0.75–0.94]) were negatively associated with daily breakfast consumption.

**FIGURE 4:**
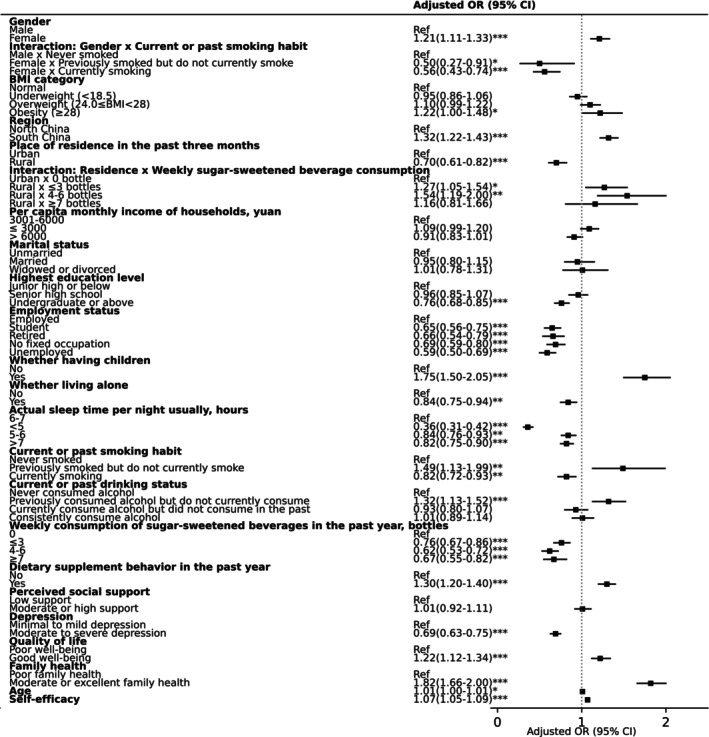
Forest plot for odds ratios of daily breakfast consumption. Ref = reference; *, **, and *** indicate statistical significance at the 0.05, 0.01, and 0.001 levels, respectively, for the *p*‐value. Northern China includes Beijing, Tianjin, Hebei, Shanxi, Inner Mongolia, Liaoning, Jilin, Heilongjiang, Shandong, Henan, Tibet, Shaanxi, Gansu, Qinghai, Ningxia and Xinjiang. Southern China encompasses Jiangsu, Anhui, Zhejiang, Shanghai, Hubei, Hunan, Jiangxi, Fujian, Yunnan, Guizhou, Sichuan, Chongqing, Guangxi, Guangdong and Hainan.

Significant interactions were observed between gender and smoking status, as well as between residence and sugar‐sweetened beverage consumption. Compared with male non‐smokers, female current and former smokers were negatively associated with daily breakfast consumption. Compared to urban residents who never consumed sugar‐sweetened beverages, rural residents consuming less than 7 bottles per week were associated with daily breakfast consumption (Figure 4).

### 3.3 Factors Associated With Daily Breakfast Consumption Stratified by Gender

**FIGURE 5:**
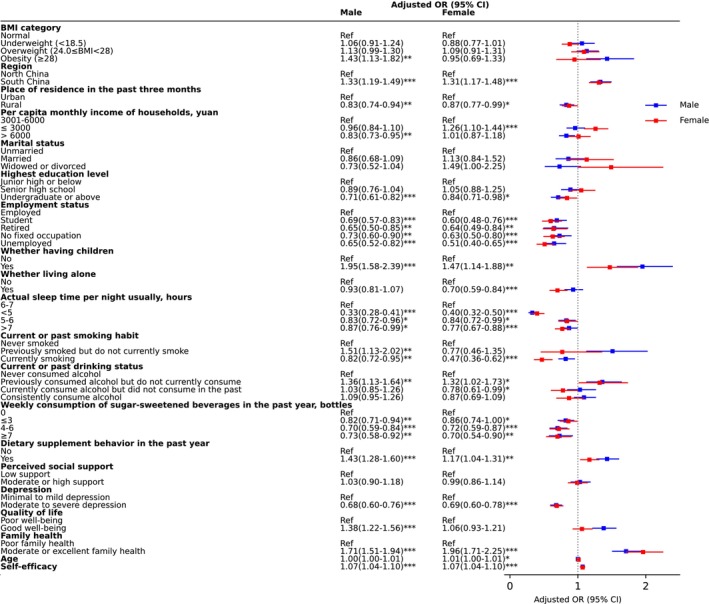
Forest plot for odd ratios of breakfast consumption regularity stratified by gender. Ref = reference; *, **, and *** indicate statistical significance at the 0.05, 0.01, and 0.001 levels, respectively, for the *p*‐value.

When stratified by gender, the results were largely consistent with the overall analysis. However, some differences emerged. In females, obesity, being a former smoker, and better quality of life were not significantly associated with daily breakfast consumption. In males, age and living alone were not significantly associated. Interestingly, higher family income was negatively associated with daily breakfast consumption in males (0.83 [0.73–0.95]) (Figure 5).

### 3.4 Factors Associated With Daily Breakfast Consumption Stratified by Residence

**FIGURE 6:**
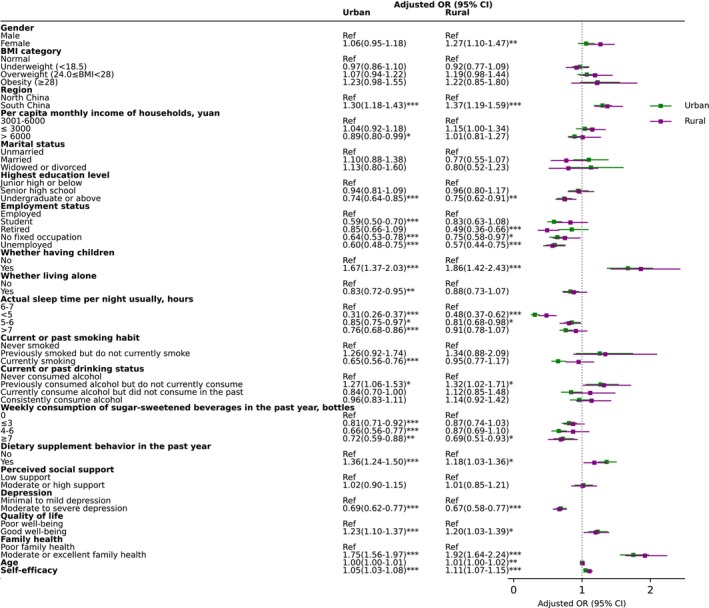
Forest plot for odd ratios of daily breakfast consumption stratified by residence. Ref = reference; *, **, and *** indicate statistical significance at the 0.05, 0.01, and 0.001 levels, respectively, for the *p*‐value.

Greater variability in associated factors was observed when stratified by place of residence. Among urban residents, female gender, being retired, and older age were not significantly associated with daily breakfast consumption. In rural residents, being a student, living alone, smoking, consuming no more than six bottles of sugar‐sweetened beverages per week, and longer sleep durations were not significant factors. For both urban and rural residents, obesity, being a former smoker, and lower family income status were not significantly associated with daily breakfast consumption. However, higher family income was negatively associated with daily breakfast consumption among urban residents (Figure 6).

## 4 Discussion

To our knowledge, this study is the first to evaluate the status and associated factors of breakfast consumption patterns at the national level in China. Through a nationwide quantitative survey, we found that 59.0% were daily breakfast consumers, while 41.0% were non‐daily breakfast consumers. For breakfast eating type pattern, food categories with the highest percentage of consumers were rice, wheat, and corn (70.5%), eggs (56.2%), and dairy products (42.0%). Of note, sociodemographic, behavioral, and health factors have been found to have an impact on breakfast consumption behaviors.

The present study has shown that over half of the Chinese population was daily breakfast consumers. The prevalence of non‐breakfast consumption was found to be higher in urban residents (non‐breakfast consumers: urban, 5.9%; rural, 7.9%), itself a predictor of low‐quality diet and unhealthy behaviors (Lenardson et al. 2015; Choi et al. 2017). Females are found to tend to be daily breakfast consumers (female, 61.1%; male, 57.0%), which is in line with findings from previous studies (Fagt et al. 2018; Drewnowski et al. 2018; Uvacsek et al. 2022). Gender differences are commonly reported in eating behaviors, since women are more likely to follow healthy dietary recommendations and maintain a healthy lifestyle (Kiefer et al. 2005; Alkazemi 2019; Chang et al. 2019). There is considerable diversity in defining the regularity of breakfast consumption among studies conducted in other countries (Fagt et al. 2018; Drewnowski et al. 2018; Gaal et al. 2018), making it difficult to compare the prevalence directly. For instance, a survey in the UK categorizes the regularity of breakfast consumption into non‐breakfast consumers (no breakfast), irregular breakfast consumers (breakfast on 1 or 2 days out of 4) and regular breakfast consumers (breakfast on 3 or 4 days out of 4) (Gaal et al. 2018), while another survey in Denmark categorizes it into skippers (breakfast 0–1 day per week), irregular consumers (2–4 breakfasts per week), and regular consumers (5–7 breakfasts per week) (Fagt et al. 2018). Our definition was based on the recommendation by the Chinese Nutrition Society in the Chinese Dietary Guidelines (2022) that the general population is advised to eat breakfast every day. Eating a good breakfast every day not only fulfills the body's energy and nutritional requirements but can also contribute to weight management, lower the risk of diabetes and cardiovascular diseases, enhance work and study productivity, and more (Guidelines TCD 2022). Moreover, the factors relevant to breakfast frequency varied when stratified by residence and gender. This finding highlights the need for region‐specific and gender‐specific strategies to promote healthy breakfast behaviors.

The breakfast eating pattern is culturally diverse across different countries, so is the choice of breakfast categories. Our study found the highest breakfast intakes of rice, wheat and corn (70.5%), representing substantial intakes of carbohydrates, is unique in China. Though previous study based on the 1989–2015 China Health and Nutrition Survey indicated that there has been a decrease in carbohydrates and an increase in protein (Huang et al. 2021), the dietary structure for breakfast is still dominated by high‐carbohydrates foods. Besides, pickled vegetables, with high content of sodium and nitrite, were present in 18.2% of Chinese breakfast. Given that sodium intake in China has been much higher than the amount recommended by the World Health Organization (Huang et al. 2021), pickled vegetables is considered as an important source of sodium and should be limited in intake. The top three categories identified for American people were breads (19.2%), cereal plus groups not including eggs (16.8%), coffee and high‐fat dessert (15.1%) (Drewnowski et al. 2018). For Mexican people, milk and sweetened breads (38%), tortillas and beans (12%), sweetened beverages (10%) are the most eaten foods at breakfast (Gibney et al. 2018). However, the varying definition of given food categories made it difficult to compare across different countries and studies (Gaal et al. 2018). It will be helpful to evaluate percentage contribution of food categories to energy and nutrients intakes at breakfast, as conducted in some surveys, to show the difference in the mean intakes of carbohydrate, total sugars, vitamins, etc. in different regions. Furthermore, the breakfast eating pattern also differs in Chinese rural and urban, female and male subgroups. For instance, people in urban area tend to have more protein intakes (e.g., egg, milk), which may be related to economic level and income (Huang et al. 2021). A previous study in Norway (Hovdenak et al. 2024) showed that individuals with low‐income are more likely to skip breakfast, with reasons of “lack of time”, “not wanting to eat,” and economic difficulties (lack of food, financial constraints), further supporting the impact of socioeconomic status on breakfast habits. The results of breakfast eating pattern suggest room for improvement in breakfast categories and quality in efforts to diminish regional and economic inequality.

Our study also suggested that eating breakfast daily is associated with better mental health, well‐being, and family health. A systematic review of 26 studies provided evidence showing that eating breakfast had positive and conclusive effects on quality of life and well‐being (Lundqvist et al. 2019). However, they highlighted the possibility that mental health might influence breakfast consumption patterns. The interplay mechanism is complex and needs further exploration.

Our study provides a comprehensive understanding of the intricate factors associated with breakfast consumption. For behavioral factors, sugar‐sweetened beverages consumption has been shown to be an important one. A study conducted in Norway showed that seldom eating breakfast was associated with higher odds for daily sugar‐sweetened beverages consumption (Skeie et al. 2019). Sugar‐sweetened beverages provide high energy intake with low nutritional value and lead to increased contributions to daily energy, which may account for the result of nutrient intake for breakfast. Clinical randomized studies suggest that sugar‐sweetened beverages may lead to suppressed appetite (Bennett et al. 2018; Poirier et al. 2019). In this sense, having breakfast may in turn decrease the chances of consuming sugar‐sweetened beverages with high calories. Similarly, nicotine and alcohol have been shown to decrease appetite and food intake via hormone regulation (Schwartz and Bellissimo 2021), which may account for the finding that individuals with current or previous smoking habits and drinking status were less likely to eat breakfast daily. However, it should be noted that the observed associations between sugar‐sweetened beverages consumption, smoking, and breakfast consumption may reflect broader dietary and lifestyle behaviors. For example, individuals who frequently consume sugar‐sweetened beverages may have an overall unhealthy diet characterized by high caloric intake and low nutritional quality. In this case, the association between sugar‐sweetened beverage consumption, smoking, and breakfast consumption could be a proxy for an overall poor dietary pattern rather than an independent effect. Previous studies indicated that there are higher odds of consuming breakfast for adolescents who had longer sleep duration, which is further verified in adolescents and adults in our study. It suggested that poor sleep health may negatively influence metabolic health (Mathew et al. 2022). For health factors, better family health indicates closer family ties and may lead to shared meals and consistent daily routines, contributing to a higher likelihood of regular breakfast consumption. On the contrary, people living alone tended to have irregular breakfast habits because of poor food accessibility and social communication (Ishikawa et al. 2018). Our study also indicates the association between depression and breakfast consumption. In consistency with our study, a recent systematic review indicated that skipping breakfast was positively associated with odds of depression, stress, and psychological distress in all age groups, underlying the importance of the impact of breakfast on mental health (Zahedi et al. 2022). However, the observational nature of the study does not allow us to establish the causal relationships between breakfast consumption and depressive symptoms. Thus, future interventional or experimental research is required to further explore the relationship. Unexpectedly, obesity was found to be positively associated with daily breakfast consumption. Though skipping breakfast is often linked to weight gain, a few studies have found a positive association between breakfast consumption and higher BMI, in alignment with our finding (Timlin and Pereira 2007). This may be due to reverse causality, where obese individuals are more likely to eat breakfast to control weight (Timlin and Pereira 2007). For sociodemographic factors, multiple factors (e.g., age, gender, region, having children, income, education levels) have an impact on breakfast consumption patterns. Among these, the effect of having children seems to be most pronounced, since people usually feel motivated to make breakfast for and take breakfast with their children during adolescence and young adulthood, and the habit of eating breakfast developed during this period may persist into the whole life. Daily breakfast consumption was associated with higher education level and employment status, themselves indicators of higher‐quality diets and improved health (Drewnowski and Specter 2004; Li and Wu 2022). Besides, it is noted that differences in factors exist between male and female, urban and rural subgroups. Our findings emphasize multiple factors in shaping breakfast behaviors, highlighting the need for multifaceted interventions to promote healthier eating habits.

The results of this study can provide some references and feasible suggestions on good habits of eating breakfast and promote healthy behaviors. However, there are limitations to the current study that must be acknowledged. Firstly, our analysis was based on self‐reported data and may cause some bias. For example, the PHQ‐9 relied on individuals' self‐report of their depressive symptoms over the past 2 weeks, which posed challenge to memory and subjective interpretation. Moreover, social desirability is a critical concern for self‐reported data. Individuals may overreport healthier eating habit for underreport skipping breakfast to align with perceived social norms. Future research should consider combining self‐reported assessments with other objective measures. Although self‐reported information may not always accurately depict actual breakfast consumption patterns, it's important to note that a majority of the world's representative population‐based dietary intake data rely on self‐reported information. Secondly, data collection was based on 1‐year recalls of participants, which is subject to memory bias. In the context of dietary intake, this bias may impact the reliability and validity of the reported data. The recalls based on a 4‐day diet as conducted in the UK may help diminish memory bias (Gaal et al. 2018). Future studies can shorten recall periods and optimize questionnaire design to reduce recall bias. Thirdly, as with any observational study, it is important to recognize that the associations identified do not imply causation, and other unmeasured factors may influence breakfast consumption patterns. Fourthly, although we listed food categories, we did not explicitly assess nutrient intake for breakfast. This lack of detailed nutritional analysis may limit the interpretation of our findings related to dietary patterns and their potential impacts. Future research should incorporate a more thorough assessment of nutrient intake to provide a comprehensive understanding of dietary quality. Lastly, the COVID‐19 pandemic has had a profound impact on daily routines and dietary habits (Di Renzo et al. 2020; Parker et al. 2023; Bennett et al. 2021). Longitudinal studies could provide valuable insights into the long‐term effects of the pandemic on breakfast consumption.

## 5 Conclusions

This nationwide study revealed that 59.0% of Chinese residents were daily breakfast consumers, while 41.0% were non‐daily breakfast consumers, with notable variations by gender and residence (urban vs. rural). The most commonly consumed breakfast food categories included staples such as rice, wheat, and corn, followed by eggs and dairy products. Our findings highlight the influence of multiple sociodemographic, behavioral, and health factors on breakfast consumption patterns, emphasizing the complexity of dietary behaviors in China. Targeted, region‐ and subgroup‐specific strategies are needed to address disparities in breakfast consumption and promote healthier breakfast habits, ultimately contributing to improved overall health outcomes.

## Author Contributions

**Ming Liu:** conceptualization (lead), formal analysis (lead), methodology (lead), project administration (lead), validation (lead), visualization (lead), writing – original draft (lead), writing – review and editing (lead). **Shujie Dong:** conceptualization (equal), formal analysis (equal), methodology (supporting), writing – original draft (equal), writing – review and editing (equal). **Yifan Li:** formal analysis (equal), software (lead), validation (equal), writing – original draft (supporting), writing – review and editing (supporting). **Shaolin Liang:** writing – review and editing (equal). **Chun Kai Leung:** writing – review and editing (equal). **Casper J. P. Zhang:** writing – review and editing (equal). **Sicun Li:** writing – review and editing (supporting). **Yibo Wu:** data curation (lead), resources (equal). **Wai‐kit Ming:** resources (lead), writing – review and editing (equal).

## Ethics Statement

The study received ethical approval from the Ethics Committee of the Health Culture Research Center of Shaanxi, China (Approval No. JKWH‐2022‐02).

## Consent

All participants were required to provide informed consent before participation.

## Conflicts of Interest

The authors declare no conflicts of interest.

## Supporting information


Appendix S1.

## Data Availability

The data utilized in this study will be made available upon prompt request.
