# Supplementary material for: Status and Associated Factors of Breakfast Consumption Among Chinese Residents: A National Cross‐Sectional Study
Source: Food Sci Nutr. 2025 May 26;13(6):e70136. doi: 10.1002/fsn3.70136 (PMC12106057; doi:10.1002/fsn3.70136)
Supplement: Supplementary file 1 — Appendix S1. [file FSN3-13-e70136-s001.docx]

**Supplementary appendix**

**Note 1:**

Questionnaires with inconsistent logical examination results, including:

Question 3 selected "male," while Question 32 selected "female reproductive tumor."

Question 4 selected "12-17 years old," while Question 11 selected "leave/retirement."

Question 4 selected "12-17 years old," while Question 10 selected "member of the Communist Party of China (CPC)" or "member of the Communist Party of China" from other parties.

Question 4 selected "12-17 years old," while Question 57 selected "married," "divorced," or "widowed."

Question 6 selected "have religious belief," while Question 10 selected "CPC member" or "CPC probationary member" or "Communist Youth League member."

Question 7 and Question 8, those with abnormal Body Mass Index values after calculation.

Table S1. Standard scales used in the questionnaire.

| **Scale** | **Measurement** | **Score** | **Cronbach’s α** |
| --- | --- | --- | --- |
| Patient Health Questionnaire-9 terms (PHQ-9) | Depression | Respondents choose 0-3 (never to nearly every day) in each item in PHQ-9 corresponding to a specific symptom of depression. Total higher scores indicate more severe depression symptoms. | 0.921 |
| 5-item World Health Organization Well-Being Index (WHO-5) | Quality of life | The scale comprises five positively worded items, each rated on a 6-point Likert scale ranging from 0 (never before) to 5 (all the time), capturing feelings over the past two weeks. | 0.950 |
| 3-item short form of Perceived Social Support Scale (PSSS-SF3) | Perceived social support | Respondents rate each item on a 7-point Likert scale, ranging from 1 (strongly disagree) to 7 (strongly agree). Total score of 3-9 represents low perceived social support, 10 to 15 shows moderate perceived social support and 16 to 21 indicates high perceived social support. | 0.888 |
| 3-item short form of New General Self-efficacy scale (NGSES-SF3) | Self-efficacy | Respondents rated each item on a 5-point Likert scale, from 1 (strongly disagree) to 5 (strongly agree), reflecting their perception of coping abilities and self-confidence. The scale yields total scores ranging from 3 to 15 points, with higher scores indicating greater self-efficacy. | 0.926 |
| Family Health Scale-Short Form (FHS-SF) | Family health | Responses of 4 or higher (indicating agreement or strong agreement) were scored as 1 and responses lower than 4 (neutrality or disagreement with the statement) received a score of 0. Items were then summed so that each participant could have a final family health score between 0 and 10 points, with higher scores indicating higher levels of family health. For clinical cutoffs, scores of < 6 points indicated poor family health, scores of 6–8 indicated moderate family health, and scores of 9 or 10 indicated excellent family health. | 0.825 |

Table S2. The Factors Associated with Breakfast Eating Behavior.

| Category | Variable |
| --- | --- |
| Socio-Demographic Factors | Age |
|  | Gender |
|  | Region |
|  | Place of residence in the past three months |
|  | Per capita monthly income of households |
|  | Marital status |
|  | Highest education level |
|  | Employment status |
|  | Whether having children |
|  | Whether living alone |
| Behavioral Factors | Actual sleep time per night usually, hours |
|  | Current or past smoking habit |
|  | Current or past drinking status |
|  | Weekly consumption of sugar-sweetened beverages in the past year |
|  | Dietary supplement behavior in the past year |
| Health Factors | BMI category |
|  | Depression |
|  | Quality of life |
|  | Family health |
|  | Perceived social support |
|  | Self-efficacy |

**Table S3. STROBE-nut: An extension of the STROBE statement for nutritional epidemiology**

Lachat C et al. (2016) STrengthening the Reporting of OBservational studies in Epidemiology – Nutritional Epidemiology (STROBE-nut): an extension of the STROBE statement. Plos Medicine 13(6) <http://dx.doi.org/10.1371/journal.pmed.1002036> [pdf](http://journals.plos.org/plosmedicine/article/asset?id=10.1371%2Fjournal.pmed.1002036.PDF) or [online](http://journals.plos.org/plosmedicine/article?id=10.1371/journal.pmed.1002036) version.

| **Item** | **Item nr** | **STROBE recommendations** | **Extension for Nutritional Epidemiology studies (STROBE-nut)** | **Reported on page #** |
| --- | --- | --- | --- | --- |
| **Title and**  **abstract** | 1 | (a) Indicate the study’s design with a commonly used term in the title or the abstract.  (b) Provide in the abstract an informative and balanced summary of what was done and what was found. | **nut-1** State the dietary/nutritional assessment method(s) used in the title, abstract, or keywords. | **Page 1-2** |
| **Introduction** |  |  |  |  |
| Background rationale | 2 | Explain the scientific background and rationale for the investigation being reported. |  | Page 3-4 |
| Objectives | 3 | State specific objectives, including any pre-specified hypotheses. |  | Page 3-4 |
| **Methods** |  |  |  |  |
| Study design | 4 | Present key elements of study design early in the paper. |  | Page 10 |
| Settings | 5 | Describe the setting, locations, and relevant dates, including periods of recruitment, exposure, follow-up, and data collection. | **nut-5** Describe any characteristics of the study settings that might affect the dietary intake or nutritional status of the participants, if applicable. | **Page 10-11** |
| Participants | 6 | a) Cohort study—Give the eligibility criteria, and the sources and methods of selection of participants. Describe methods of follow-up.  Case-control study—Give the eligibility criteria, and the sources and methods of case ascertainment and control selection. Give the rationale for the choice of cases and controls.  Cross-sectional study—Give the eligibility criteria, and the sources and methods of selection of participants.  (b) Cohort study—For matched studies, give matching criteria and number of exposed and unexposed.  Case-control study—For matched studies, give matching criteria and the number of controls per case. | **nut-6** Report particular dietary, physiological or nutritional characteristics that were considered when selecting the target population. | **Page 10-11** |
| Variables | 7 | Clearly define all outcomes, exposures, predictors, potential confounders, and effect modifiers. Give diagnostic criteria, if applicable. | **nut-7.1** Clearly define foods, food groups, nutrients, or other food components.  **nut-7.2** When using dietary patterns or indices, describe the methods to obtain them and their nutritional properties. | **Page 11-12** |
| Data sources - measurements | 8 | For each variable of interest, give sources of data and details of methods of assessment (measurement).Describe comparability of assessment methods if there is more than one group. | **nut-8.1** Describe the dietary assessment method(s), e.g., portion size estimation, number of days and items recorded, how it was developed and administered, and how quality was assured. Report if and how supplement intake was assessed.  **nut-8.2** Describe and justify food composition data used. Explain the procedure to match food composition with consumption data. Describe the use of conversion factors, if applicable.  **nut-8.3** Describe the nutrient requirements, recommendations, or dietary guidelines and the evaluation approach used to compare intake with the dietary reference values, if applicable.  **nut-8.4** When using nutritional biomarkers, additionally use the STROBE Extension for Molecular Epidemiology (STROBE-ME). Report the type of biomarkers used and their usefulness as dietary exposure markers.  **nut-8.5** Describe the assessment of nondietary data (e.g., nutritional status and influencing factors) and timing of the assessment of these variables in relation to dietary assessment.  **nut-8.6** Report on the validity of the dietary or nutritional assessment methods and any internal or external validation used in the study, if applicable. | **Page 11-12** |
| Bias | 9 | Describe any efforts to address potential sources of bias. | **nut-9** Report how bias in dietary or nutritional assessment was addressed, e.g., misreporting, changes in habits as a result of being measured, or data imputation from other sources | **Page 10** |
| Study Size | 10 | Explain how the study size was arrived at. |  | Page 10 |
| Quantitative variables | 11 | Explain how quantitative variables were handled in the analyses. If applicable, describe which groupings were chosen and why. | **nut-11** Explain categorization of dietary/nutritional data (e.g., use of N-tiles and handling of nonconsumers) and the choice of reference category, if applicable. | **Page 11-12** |
| Statistical  Methods | 12 | (a) Describe all statistical methods, including those used to control for confounding  (b) Describe any methods used to examine subgroups and interactions.  (c) Explain how missing data were addressed.  (d) Cohort study—If applicable, explain how loss to follow-up was addressed.  Case-control study—If applicable, explain how matching of cases and controls was addressed.  Cross-sectional study—If applicable, describe analytical methods taking account of sampling strategy.  (e) Describe any sensitivity analyses. | **nut-12.1** Describe any statistical method used to combine dietary or nutritional data, if applicable.  **nut-12.2** Describe and justify the method for energy adjustments, intake modeling, and use of weighting factors, if applicable.  **nut-12.3** Report any adjustments for measurement error, i.e,. from a validity or calibration study. | **Page 13** |
| **Results** |  |  |  |  |
| Participants | 13 | (a) Report the numbers of individuals at each stage of the study—e.g., numbers potentially eligible, examined for eligibility, confirmed eligible, included in the study, completing follow-up, and analyzed.  (b) Give reasons for non-participation at each stage.  (c) Consider use of a flow diagram. | **nut-13** Report the number of individuals excluded based on missing, incomplete or implausible dietary/nutritional data. | **Page 4** |
| Descriptive data | 14 | (a) Give characteristics of study participants (e.g., demographic, clinical, social) and information on exposures and potential confounders  (b) Indicate the number of participants with missing data for each variable of interest  (c) Cohort study—Summarize follow-up time (e.g., average and total amount) | **nut-14** Give the distribution of participant characteristics across the exposure variables if applicable. Specify if food consumption of total population or consumers only were used to obtain results. | **Page 4-5** |
| Outcome data | 15 | Cohort study—Report numbers of outcome events or summary measures over time.  Case-control study—Report numbers in each exposure category, or summary measures of exposure.  Cross-sectional study—Report numbers of outcome events or summary measures. |  | **Page 4-5** |
| Main results | 16 | (a) Give unadjusted estimates and, if applicable, confounder-adjusted estimates and their precision (e.g., 95% confidence interval).  Make clear which confounders were adjusted for and why they were included.  (b) Report category boundaries when continuous variables were categorized.  (c) If relevant, consider translating estimates of relative risk into absolute risk for a meaningful time period. | **nut-16** Specify if nutrient intakes are reported with or without inclusion of dietary supplement intake, if applicable. | **Page 5-6** |
| Other analyses | 17 | Report other analyses done—e.g., analyses of subgroups and interactions and sensitivity analyses. | **nut-17** Report any sensitivity analysis (e.g., exclusion of misreporters or outliers) and data imputation, if applicable. | **Page 6** |
| **Discussion** |  |  |  |  |
| Key results | 18 | Summarize key results with reference to study objectives. |  | Page 7-8 |
| Limitation | 19 | Discuss limitations of the study, taking into account sources of potential bias or imprecision. Discuss both direction and magnitude of any potential bias. | **nut-19** Describe the main limitations of the data sources and assessment methods used and implications for the interpretation of the findings. | **Page 9** |
| Interpretation | 20 | Give a cautious overall interpretation of results considering objectives, limitations, multiplicity of analyses, results from similar studies, and other relevant evidence. | **nut-20** Report the nutritional relevance of the findings, given the complexity of diet or nutrition as an exposure. | **Page 7-9** |
| Generalizability | 21 | Discuss the generalizability (external validity) of the study results. |  | **Page 9** |
| **Other information** |  |  |  |  |
| Funding | 22 | Give the source of funding and the role of the funders for the present study and, if applicable, for the original study on which the present article is based. |  | **Page 18** |
| *Ethics* |  |  | **nut-22.1** Describe the procedure for consent and study approval from ethics committee(s). | **Page 18** |
| *Supplementary material* |  |  | **nut-22.2** Provide data collection tools and data as online material or explain how they can be accessed. | **Supplementary material** |


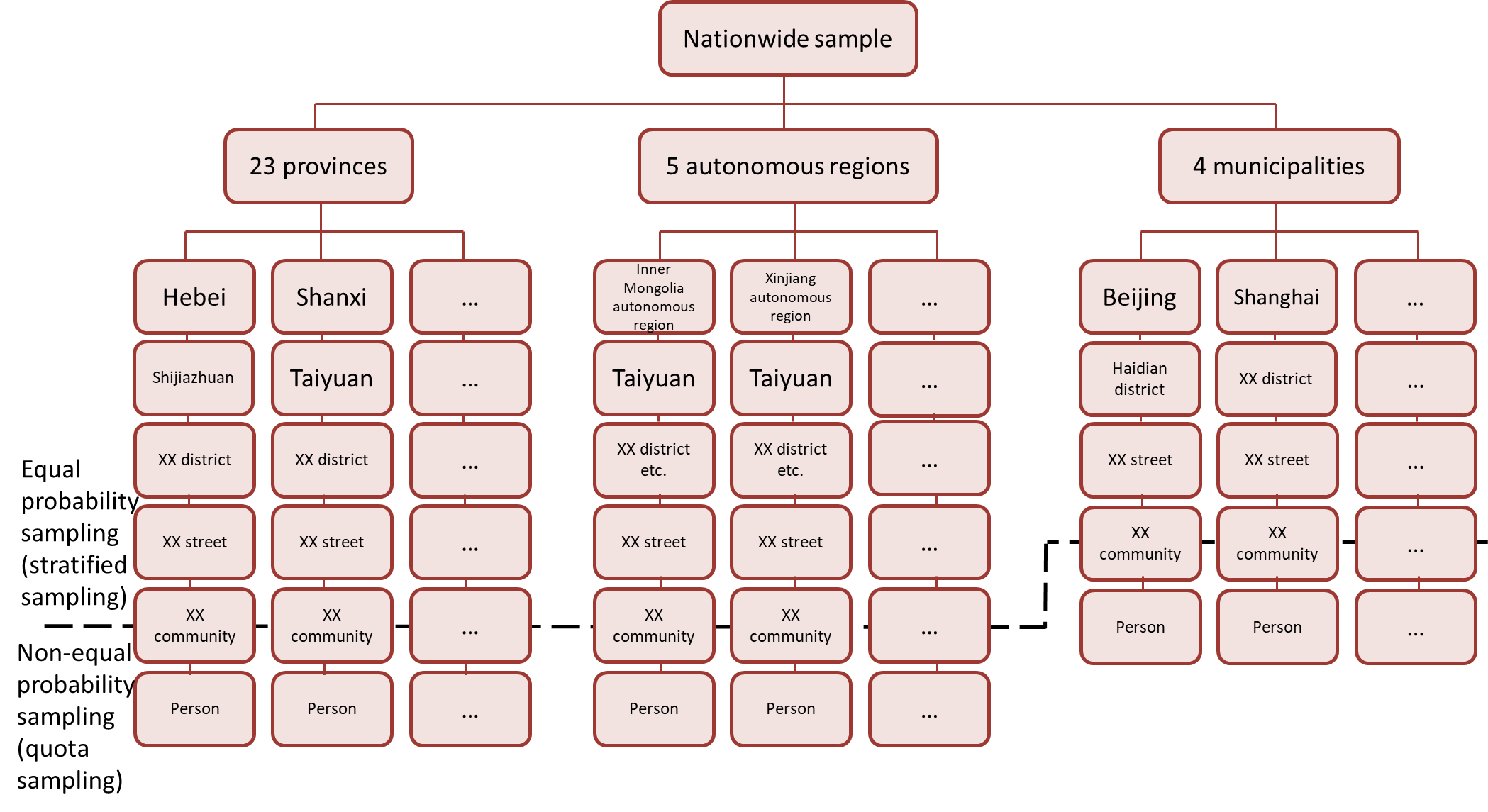
Fig. S1 Flowchart of sampling method

**
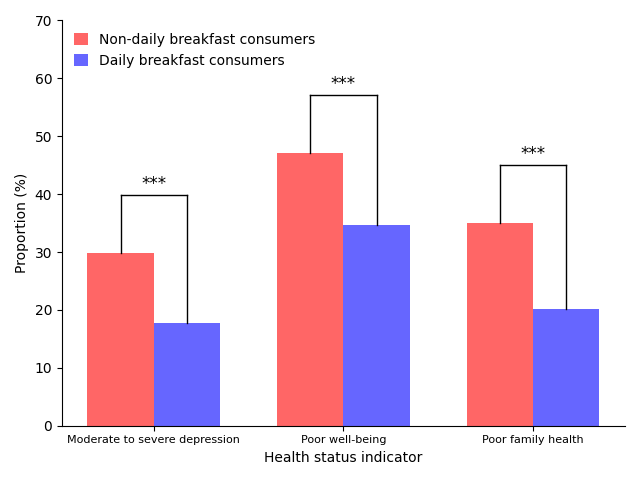
**Fig. S2. Comparison of health status indicators for daily and non-daily breakfast consumers. Proportions of moderate to severe depression, poor well-being, poor family health were calculated using PHQ-9, WHO-5 and FHS-SF. Statistically significant differences (p < 0.001=***) determined using Chi-squared test.


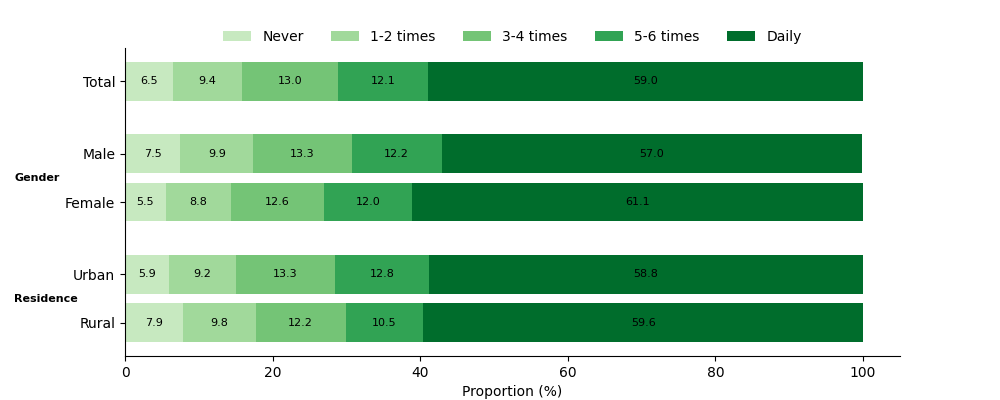


Fig. S3. Comparison of gender and residence factors associated with breakfast eating frequency.


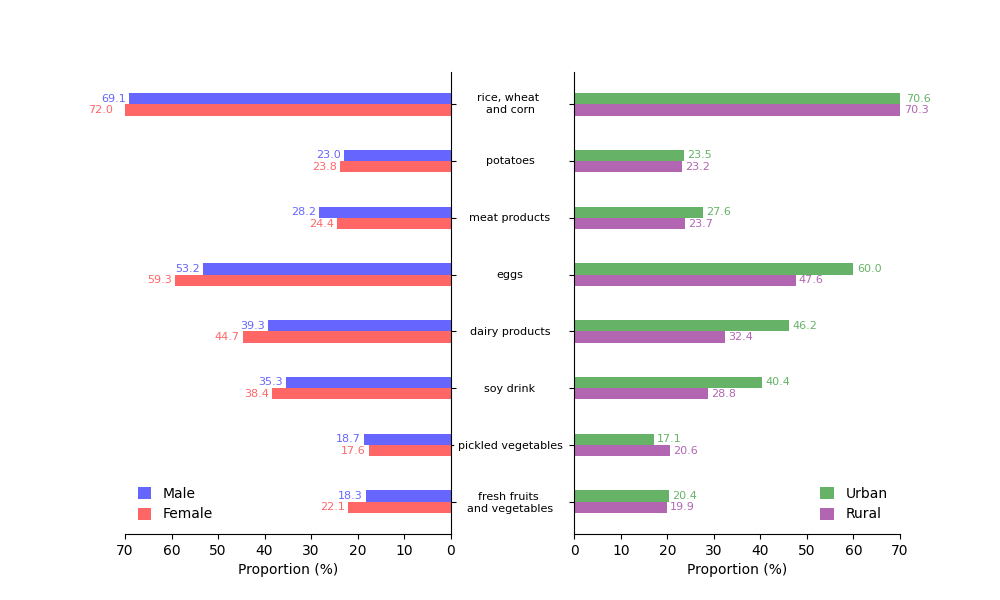
Fig. S4. Comparison of the proportion of food categories in daily breakfast by gender and residence.
